# Metabolic characteristics of programmed cell death‐ligand 1‐expressing lung cancer on ^18^F‐fluorodeoxyglucose positron emission tomography/computed tomography

**DOI:** 10.1002/cam4.1215

**Published:** 2017-10-04

**Authors:** Kazuki Takada, Gouji Toyokawa, Tatsuro Okamoto, Shingo Baba, Yuka Kozuma, Taichi Matsubara, Naoki Haratake, Takaki Akamine, Shinkichi Takamori, Masakazu Katsura, Fumihiro Shoji, Hiroshi Honda, Yoshinao Oda, Yoshihiko Maehara

**Affiliations:** ^1^ Department of Surgery and Science Graduate School of Medical Sciences Kyushu University Fukuoka Japan; ^2^ Department of Anatomic Pathology Graduate School of Medical Sciences Kyushu University Fukuoka Japan; ^3^ Department of Clinical Radiology Graduate School of Medical Sciences Kyushu University Fukuoka Japan

**Keywords:** FDG, lung cancer, PD‐L1, PET/CT, SUVmax

## Abstract

Programmed cell death‐1 (PD‐1) and programmed cell death‐ligand 1 (PD‐L1) have been identified as novel targets of immunotherapy of lung cancer. In present study, we evaluated the metabolic characteristics of lung cancer by using ^18^F‐fluorodeoxyglucose positron emission tomography/computed tomography (^18^F‐FDG PET/CT) with regard to PD‐L1 protein expression. PD‐L1 protein expression was evaluated by immunohistochemistry with the antibody clone SP142 in 579 surgically resected primary lung cancer patients. Cases with less than 5% tumor membrane staining were considered negative. We examined the association between the frequency of PD‐L1 protein expression and the maximum standardized uptake value (SUVmax) in preoperative ^18^F‐FDG PET/CT. The cut‐off values for SUVmax were determined by receiver operating characteristic curve analyses. The SUVmax was significantly higher in nonsmall cell lung cancer (NSCLC) patients with PD‐L1 protein expression compared with those without PD‐L1 protein expression (*P *<* *0.0001). However, there was no correlation between SUVmax and PD‐L1 protein expression in patients with neuroendocrine tumors (*P *=* *0.6545). Multivariate analysis revealed that smoking, the presence of pleural invasion, and high SUVmax were independent predictors of PD‐L1 positivity. PD‐L1‐expressing NSCLC had a high glucose metabolism. The SUVmax in preoperative ^18^F‐FDG PET/CT was a predictor of PD‐L1 protein expression in patients with NSCLC.

## Introduction

Lung cancer is the leading cause of cancer‐related death worldwide 1. Recently, molecular targeted therapy has greatly improved the clinical course of patients with nonsmall cell lung cancer (NSCLC) with common driver mutations, including mutations in the *epidermal growth factor receptor* (*EGFR*) and translocation in the *anaplastic lymphoma kinase* (*ALK*) genes 2. Despite advances in therapies, the prognosis of patients with NSCLC without driver oncogene mutations remains poor 3.

Programmed cell death‐1 (PD‐1)/programmed cell death‐ligand 1 (PD‐L1) targeted immunotherapy improved the prognoses of various cancers 4, 5. PD‐1, a member of the immunoglobulin superfamily B7, is expressed on the surface of T cells, and regulates T cell activation 4. PD‐L1, the ligand of PD‐1, is expressed by many cancers, including NSCLC, and might aid the evasion of antitumor immune responses at the tumor site 4, 6, 7. Recent clinical trials of refractory NSCLC showed that an antiPD‐1 antibody, Nivolumab, yielded significant survival benefits 8, 9, and that antiPD‐1 therapy is the standard treatment for NSCLC patients after the failure of first‐line chemotherapy. The expression of PD‐L1 protein in tumor cells is expected to be a prognostic and predictive biomarker for responses to antiPD‐1/PD‐L1 antibodies in lung cancer 10, 11. Subset analysis of CheckMate‐057 showed a close correlation between PD‐L1 protein expression and the efficacy of PD‐1 antibodies 9. Therefore, it is important to examine PD‐L1 protein expression as well as the specific genotypes of *EGFR* mutations and *ALK* rearrangements in patients.

^18^F‐fluorodeoxyglucose positron emission tomography/computed tomography (^18^F‐FDG PET/CT) scan is an essential imaging modality in diagnosis and staging of lung cancer 12, 13. Glucose metabolism in cancer tissues measured on ^18^F‐FDG PET/CT, is a significant biomarker to characterize cancers. Correlations between FDG uptake and biologic features of cancers such as proliferation, histologic type, tumor differentiation, and hypoxia were reported 14, 15, 16, 17, 18, 19. Moreover, associations between glucose metabolism and *EGFR* mutations/*ALK* rearrangements were also reported 20, 21. However, the relationship between glucose metabolism and PD‐L1 protein expression is unclear. We recently revealed the Ki‐67 index in PD‐L1‐positive patients was higher than in PD‐L1‐negative patients in the analysis of 205 patients with squamous cell carcinoma (SCC) 22. Furthermore, PD‐L1 expression was significantly correlated with increased Ki‐67 labeling index by multivariate analysis in patients with adenocarcinoma (ADC) 23 and significant differences in FDG uptake across histological subtypes and differentiation groups in NSCLC paralleled similar differences in the Ki‐67 index 24. Therefore, we speculated that FDG uptake might be associated with PD‐L1 protein expression in patients with lung cancer.

This translational study examined PD‐L1 protein expression in primary lung cancer patients who had undergone surgical resection and investigated the association between PD‐L1 protein expression and the maximum standardized uptake value (SUVmax) in preoperative ^18^F‐FDG PET/CT.

## Materials and Methods

### Patients and samples

We retrospectively examined patients with primary lung cancer who underwent complete surgical resection at the Department of Surgery and Science, Graduate School of Medical Sciences, Kyushu University. Among them, we selected patients who were investigated with chest CT and ^18^F‐FDG PET/CT before surgery. Four hundred and forty‐one patients with ADC and 103 patients with SCC until December 2015, and four patients with large cell carcinoma (LCC), 16 patients with small cell lung carcinoma (SCLC) and 15 patients with large cell neuroendocrine carcinoma (LCNEC) until June 2016 were included in this study. A total of 579 paraffin‐embedded specimens were retrieved from the registry of the Department of Anatomic Pathology, Graduate School of Medical Sciences, Kyushu University. Patients with a history of SCC of the head and neck or esophagus were excluded from this study because of the possibility of metastatic SCC from these cancers. Patients who received neoadjuvant therapy were also excluded because of inconsistency in the expression of PD‐L1 on tumor cells before and after neoadjuvant chemotherapy 25. Clinicopathological features, including age at surgery, gender, smoking status, pathologic tumor‐node‐metastasis stage (seventh edition of the Lung Cancer Staging System), pleural or lymphovascular invasion and SUVmax were examined. Tumor differentiation, histological subtype of ADC (World Health Organization Classification 2015), and *EGFR* mutation status were also examined by subset analysis. *EGFR* status was determined in tumor tissues using the peptide nucleic acid‐locked nucleic acid polymerase chain reaction clamp method (Mitsubishi Chemical Medience, Tokyo, Japan) in 377 specimens 26. Clinical information and follow‐up data were obtained from the patients’ medical records. We obtained informed consent from each patient, and this study was approved by our institutional review board (Kyushu University, IRB No. 28‐100).

### Chest CT

Chest CT scanning was performed in a supine position during inspiratory breath‐hold using various multi‐detector row scanners: Aquilion 4 (Toshiba), Aquilion 64 (Toshiba), Aquilion ONE (Toshiba), Aquilion ONE Vision (Toshiba), SOMATOM Plus4 Volume Zoom (Siemens), Briliance CT (Phillips), and Briliance iCT (Phillips). Imaging parameters for thin‐section CT were as follows: tube voltage 120 kVp; tube current 100–500 mA; scan field of view 320–360 mm; and slice thickness 2 mm. A real exposure control (Toshiba) of automatic exposure control (Siemens and Phillips) was added in each study. All CT data sets were transferred to a Picture Archiving and Communication System, which was accessible for workstations (Volume Analyzer, Synapse‐Vincent, Fujifilm, Tokyo) with a specialized application for the lungs.

## ^18^F‐FDG PET/CT

In each patient, 185 MBq FDG was intravenously administered after fasting for at least 4 h. Scans were conducted from the middle of the thigh to the top of the skull 60 min after FDG administration. FDG‐PET/CT images were obtained by integrated PET/CT scanner (Discovery STE; GE Medical Systems, Milwaukee, WI) or Biograph mCT (Siemens Medical Solutions, Erlangen, Germany). All emission scans were performed in three‐dimensional mode, and acquisition time per bed position was 3 min for Discovery STE and 2 min for Biograph mCT. We reconstructed PET images using the ordered‐subset expectation–maximization method (VUE Point Plus) with two full iterations of 28 subsets for the Discovery STE and iterative True‐X algorithm and TOF (Ultra HD‐PET) with two full iterations of 21 subsets. The True‐X algorithm incorporates an additional specific correction for the point‐spread function. The full‐width at half‐maximum values of the Discovery STE and Biograph mCT were 5.2 and 4.4 mm, respectively. A low‐dose 16‐slice CT (tube voltage 120 kV; effective tube current 30–250 mA, Discovery STE) and a low‐dose 32‐slice CT (tube voltage 120 kV; use of angular and longitudinal dose modulation, CAREDose4D^®^, Biograph mCT) from the vertex to the proximal thigh were performed for attenuation correction, and for determining the precise anatomic location of lesions before acquisition of PET images. CT scans were reconstructed by filtered back projection into 512  ×  512 pixel images with slice thickness of 5 mm to match the PET scan. FDG uptake in lesions was evaluated using SUVmax, calculated by dedicated workstation for each scanner.

### Immunohistochemical analysis

Immunohistochemistry was performed in 579 cases of surgically resected primary lung cancer using formalin‐fixed tissue sections according to our PD‐L1 immunohistochemistry protocol as described previously 27, 28.

The primary antibody was an antihuman PD‐L1 rabbit monoclonal antibody (clone SP142, dilution 1:100; Spring Bioscience, Ventana, Tucson, AZ). Carcinoma cells showing membranous staining for PD‐L1 were evaluated as positive cells. The proportion of PD‐L1‐positive cells was independently estimated as the percentage of total carcinoma cells in whole sections by three investigators (K.T., M.K., and G.T.). If the independent assessments did not agree, the slides were reviewed by all three investigators together to achieve consensus. The consensus judgments were adopted as the final results. Cases with <5% tumor membrane staining were considered negative in this study. Sections from human placentas were used as positive controls.

### Statistical analysis

Associations between PD‐L1 protein expression and patient characteristics were analyzed using Fisher's exact test, and univariate and multivariate analyses of the relationship between PD‐L1 protein expression and other patient characteristics were performed by logistic regression analysis with backward elimination method. Cut‐off values for SUVmax were determined by receiver operating characteristic curve analyses. We examined the association between the frequency of PD‐L1 protein expression and SUVmax in preoperative ^18^F‐FDG PET/CT using Student's *t*‐test. Correlations between the proportion of PD‐L1‐positive carcinoma cells and SUVmax in preoperative ^18^F‐FDG PET/CT were assessed using Spearman's correlation coefficient test. All statistical analyses were performed by JMP Statistical Discovery Software (v11.0; SAS Institute, Cary, NC, USA). *P* values < 0.05 were statistically significant.

## Results

### Association between PD‐L1 protein expression and SUVmax in patients with lung cancer

A total of 579 patients with primary lung cancer (441 with ADC, 103 with SCC, four with LCC, 16 with SCLC and 15 with LCNEC) who underwent complete surgical resection were included in the present study (Table 1). Three hundred and thirty‐eight (58.4%) patients were male, and 354 (61.1%) were smokers; the median age of the whole study population was 69 years (range, 36–89 years).

**Table 1:** Clinicopathological characteristics of all patients

Factors	Value or no. of patients
Age (years)	
Median	69
Range	36–89
Sex	
Male	338
Female	241
Smoking status	
Never‐smoker	225
Smoker	354
Tumor size (mm)	
Median	22
Range	4–123
T	
T1	337
T2	194
T3	39
T4	9
N	
N0	474
N1	56
N2	49
N3	0
Stage	
IA	308
IB	119
IIA	54
IIB	37
IIIA	58
IIIB	3
IV	0
pl	
Absent	445
Present	134
ly	
Absent	512
Present	67
v	
Absent	411
Present	168
Histology	
Adenocarcinoma	441
Squamous cell carcinoma	103
Large cell carcinoma	4
Small cell carcinoma	16
Large cell neuroendocrine carcinoma	15

pl, pleural invasion; ly, lymphatic invasion, v: vascular invasion.

**Figure 1:**
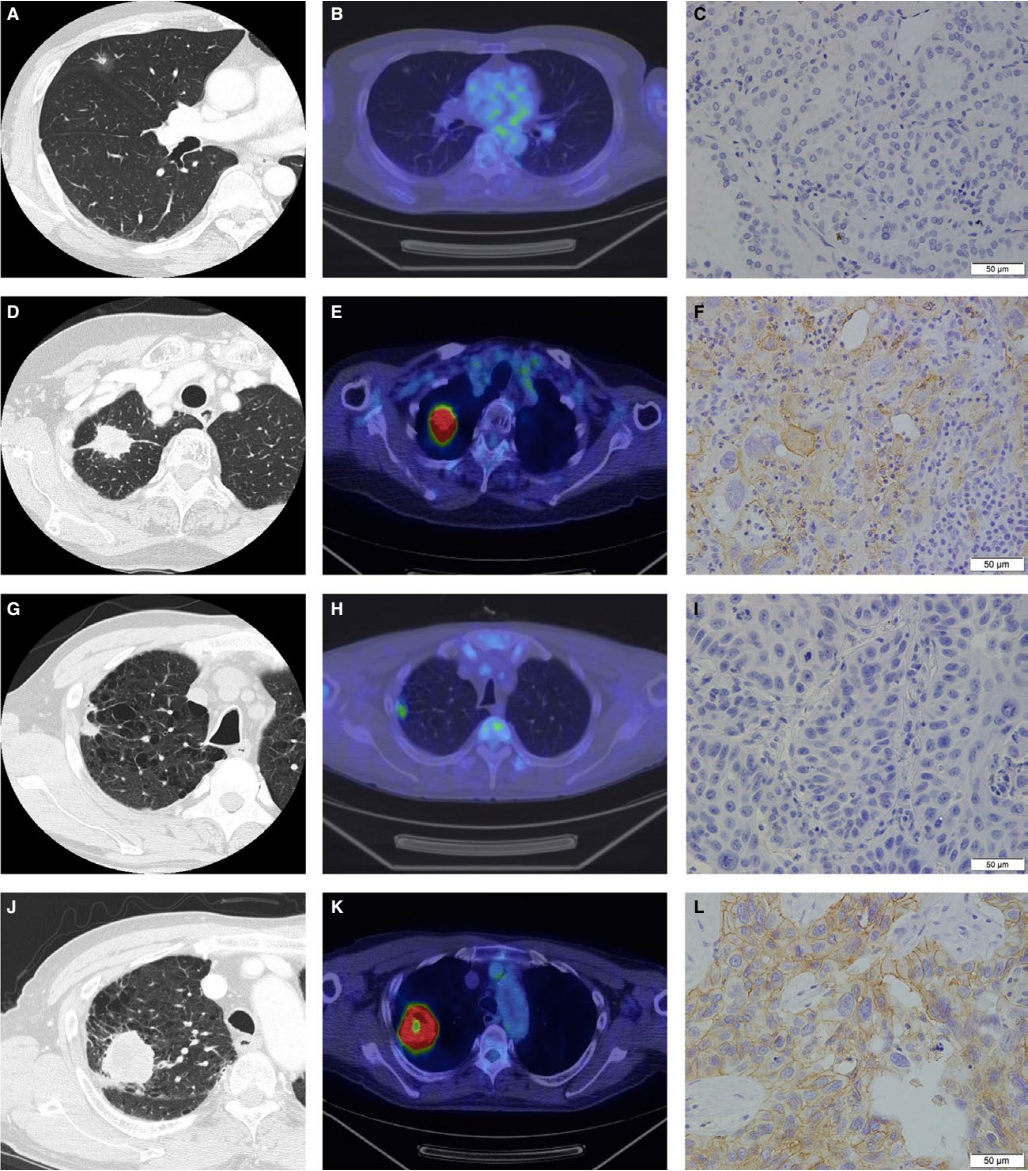
Representative images of computed tomography (CT), ^18^F‐fluorodeoxyglucose positron emission tomography/CT (^18^F‐FDG PET/CT) and immunohistochemistry in patients (A–C, G–I) without and (D–F, J–L) with programmed cell death‐ligand 1 (PD‐L1) protein expression in (A‐F) ADC and (G–L) SCC. The maximum standardized uptake values (SUVmax) are (B) 0, (E) 20.3, (H) 4.61 and (K) 22.61. ADC, adenocarcinoma; SCC, squamous cell carcinoma. Scale bar: 50 *μ*m.

**Figure 2:**
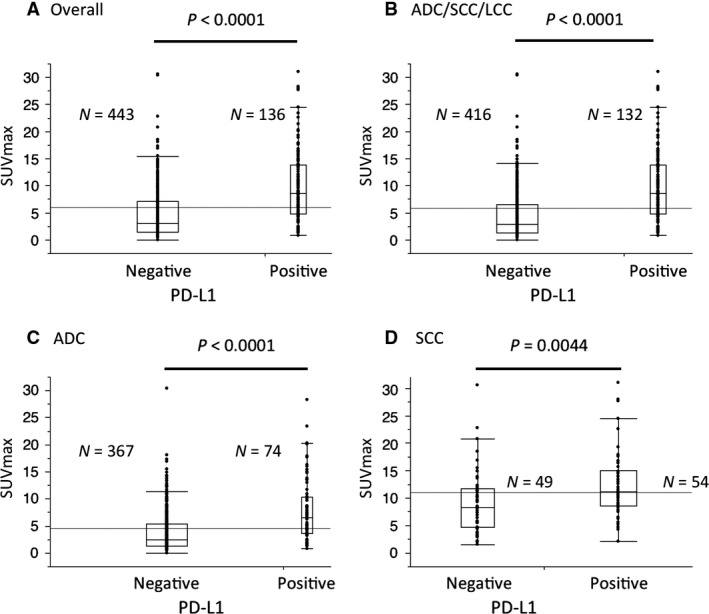
The maximum standardized uptake value (SUVmax) according to programmed cell death‐ligand 1 (PD‐L1) protein expression. The SUVmax was significantly higher in patients with PD‐L1 protein expression than those without PD‐L1 protein expression in the analyses of (A) overall (*P *<* *0.0001), (B) ADC/SCC/LCC (*P *<* *0.0001), (C) ADC (*P *<* *0.0001) and (D) SCC (*P *=* *0.0044). ADC, adenocarcinoma; SCC, squamous cell carcinoma; LCC, large cell carcinoma.

Figure 1 and Figure S1 show representative thin‐section CT, PET/CT, and immunohistochemistry staining for PD‐L1 in patients without or with PD‐L1 protein expression. Table 2 shows a summary of the frequency of PD‐L1 protein expression and SUVmax in PET/CT. The mean value of SUVmax was 5.91 (range: 0.0–31.05), 4.50 (range: 0.0–30.4), 10.97 (range: 1.50–31.05), 12.76 (range: 4.81–21.4), 7.15 (range: 2.42–14.7), and 9.29 (range: 2.80–14.57) in the analysis of overall, ADC, SCC, LCC, SCLC, and LCNEC, respectively. The percentage of tumors with positive PD‐L1 protein expression was 23.5% (136/579), 16.8% (74/441), 52.4% (54/103), 100% (4/4), 6.2% (1/16), and 20.0% (3/15) in the analysis of overall, ADC, SCC, LCC, SCLC, and LCNEC, respectively. The SUVmax was significantly higher in patients with PD‐L1 protein expression than without in the analyses of (A) overall (*P *<* *0.0001), (B) ADC/SCC/LCC (*P *<* *0.0001), (C) ADC (*P *<* *0.0001) and (D) SCC (*P *=* *0.0044) (Table 2, Fig. 2). In contrast, there was no correlation between SUVmax and PD‐L1 protein expression in neuroendocrine tumors (SCLC/LCNEC) (*P *=* *0.6545) (Figure S2). Spearman's correlation coefficient test also showed positive correlation between the proportion of PD‐L1‐positive carcinoma cells and SUVmax in the analyses of (A) overall (Spearman's rho = 0.4370, *P *<* *0.0001), (B) ADC/SCC/LCC (Spearman's rho = 0.4607, *P *<* *0.0001), (C) ADC (Spearman's rho =0.3559, *P *<* *0.0001) and (D) SCC (Spearman's rho =0.2628, *P *=* *0.0073) (Figure S3).

**Table 2:** Summary of the frequency of PD‐L1 protein expression and SUVmax in preoperative ^18^F‐FDG PET/CT

Histology	*N*	SUVmax, mean value (range)	PD‐L1, *N* (%)		SUVmax according to PD‐L1 expression, mean value (range)		
			Negative	Positive	Negative	Positive	*P* value
Overall^a^	579	5.91 (0–31.05)	443 (76.5)	136 (23.5)	4.69 (0–30.6)	9.89 (0.8–31.05)	<0.0001
ADC	441	4.50 (0–30.4)	367 (83.2)	74 (16.8)	3.84 (0–30.4)	7.81 (0.8–28.3)	<0.0001
SCC	103	10.97 (1.5–31.05)	49 (47.6)	54 (52.4)	9.18 (1.5–30.6)	12.60 (2.1–31.05)	0.0044
LCC	4	12.76 (4.81–21.4)	0 (0)	4 (100)	‐	12.76 (4.81–21.4)	‐
SCLC	16	7.15 (2.42–14.7)	15 (93.8)	1 (6.2)	7.40 (2.42–14.7)	3.4	0.3225
LCNEC	15	9.29 (2.8–14.57)	12 (80.0)	3 (20.0)	8.88 (2.8–14.57)	10.91 (6.1–14.54)	0.4491

PD‐L1, programmed cell death‐ligand 1; SUVmax, the maximum standardized uptake value; ^18^F‐FDG PET/CT, ^18^F‐fluorodeoxyglucose positron emission tomography/computed tomography; ADC, adenocarcinoma; SCC, squamous cell carcinoma; LCC, large cell carcinoma; SCLC, small cell carcinoma; LCNEC, large cell neuroendocrine carcinoma.

a : ADC, SCC, LCC, SCLC and LCNEC.

### Univariate and multivariate analyses of the relationship between PD‐L1 protein expression and other patient characteristics

**Figure 3:**
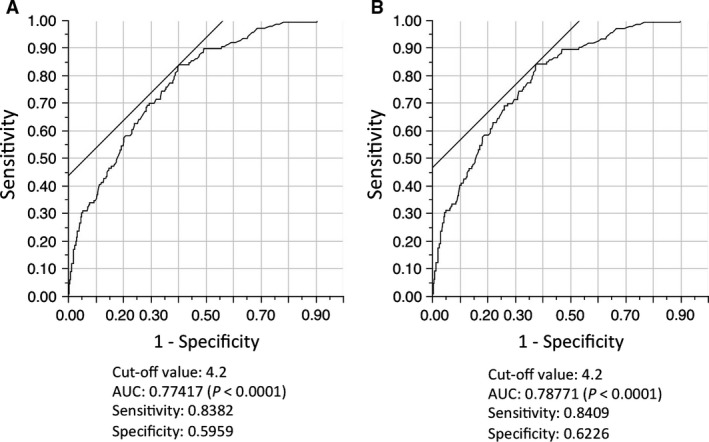
Representative images of receiver operating characteristic (ROC) curves in the analyses of (A) overall and (B) ADC/SCC/LCC. ADC, adenocarcinoma; SCC, squamous cell carcinoma; LCC, large cell carcinoma; AUC, area under curve.

We examined the association between PD‐L1 protein expression and other patient characteristics, especially SUVmax. We determined the preferable SUVmax cut‐off level using receiver operating characteristic curve analyses (Fig. 3, Figure S4). Multivariate analysis revealed that SUVmax in PET/CT was a predictor of PD‐L1 protein expression in patients with lung cancer, especially NSCLC (Table 3). Additionally, smoking and the presence of pleural invasion were also predictors of PD‐L1 protein expression. In the subset analysis of ADC and SCC, PD‐L1 protein expression was also higher in patients with high SUVmax (Tables S1 S2). Table S3 shows the frequency of PD‐L1 protein expression according to smoking history and SUVmax in PET/CT. The frequency of PD‐L1 protein expression was very high in patients with smoking history (or ≥30 pack years in cases of SCC because almost all patients with SCC were smokers) and high SUVmax.

**Table 3:** Univariate and multivariate analyses of the relation between PD‐L1 protein expression and other patient characteristics

Factors	Overall^a^				ADC/SCC/LCC			
	Univariate analysis		Multivariate analysis		Univariate analysis		Multivariate analysis	
	OR (95%CI)	*P* value	OR (95%CI)	*P* value	OR (95%CI)	*P* value	OR (95%CI)	*P* value
Age (years)								
≥69/<69	1.15 (0.78–1.69)	0.4801			1.03 (0.69–1.52)	0.8942		
Sex								
Male/Female	3.77 (2.41–6.08)	<0.0001			3.89 (2.48–6.29)	<0.0001		
Smoking status								
Smoker/Never‐smoker	4.67 (2.89–7.90)	<0.0001	3.50 (2.10–6.05)	<0.0001	4.94 (3.04–8.38)	<0.0001	3.85 (2.29–6.72)	<0.0001
Stage								
≥II/I	2.42 (1.60–3.65)	<0.0001			2.73 (1.79–4.17)	<0.0001		
pl								
Present/Absent	3.43 (2.25–5.22)	<0.0001	1.77 (1.11–2.83)	0.0167	3.98 (2.58–6.16)	<0.0001	2.01 (1.23–3.28)	0.0054
ly								
Present/Absent	1.12 (0.61–1.98)	0.7010			1.37 (0.72–2.51)	0.3260		
v								
Present/Absent	3.15 (2.11–4.72)	<0.0001			3.78 (2.49–5.74)	<0.0001		
SUVmax^b^								
High/Low	7.64 (4.75–12.81)	<0.0001	5.46 (3.29–9.40)	<0.0001	8.72 (5.35–14.82)	<0.0001	6.15 (3.66–10.75)	<0.0001

PD‐L1, programmed cell death‐ligand 1; ADC, adenocarcinoma; SCC, squamous cell carcinoma; LCC, large cell carcinoma; OR, odds ratio; CI, confidence interval; pl, pleural invasion; ly, lymphatic invasion; v, vascular invasion; SUVmax, the maximum standardized uptake value.

a ADC, SCC, LCC, SCLC and LCNEC.

b cut‐off values are 4.2 and 4.2 in analyses of overall and ADC/SCC/LCC, respectively.

## Discussion

We examined the relationship between PD‐L1 protein expression and other patient characteristics. Glucose metabolism was generally higher in patients with PD‐L1 protein expression than those without PD‐L1. Additionally, multivariate analysis revealed that smoking, the presence of pleural invasion, and high SUVmax in PET/CT were predictors of PD‐L1 protein expression in patients with lung cancer, especially NSCLC. Glucose metabolism of cancer cells measured on ^18^F‐FDG PET/CT is a significant biomarker for metabolic characteristics of cancer cells, and correlates with important features such as proliferation, histologic type, tumor differentiation, and hypoxia 14, 15, 16, 17, 18, 19. Moreover, the correlation between glucose metabolism and *EGFR* mutations/*ALK* rearrangements was also reported 20, 21. Recently, a correlation between metabolic information of FDG‐PET and tissue expression of immune markers in patients with NSCLC who are candidates for upfront surgery was reported 29. In that paper, they found a statistically significant correlation between SUVmax and SUVmean with the expression of CD8‐tumor infiltrating lymphocytes (TILs) and PD‐1‐TILs, but no correlation between SUVmax and SUVmean with PD‐L1 tumor expression. We hypothesize they might find a positive correlation between SUVmax and SUVmean with PD‐L1 tumor expression if they performed a larger analysis. To the best of our knowledge, the present report is the first to show a statistically significant association between metabolic imaging parameter SUVmax and PD‐L1 tumor protein expression in surgically resected lung cancer. PD‐L1‐expressing NSCLC demonstrated high SUVmax, suggesting PD‐L1 protein expression is related to malignant features with high glucose metabolism.

Although ^18^F‐FDG PET/CT is valuable for evaluating PD‐L1 protein expression in NSCLC, a mechanistic link between FDG uptake and PD‐L1 protein expression remains unclear. The mechanism of FDG uptake within lung cancer cells involves glucose metabolism, hypoxia, angiogenesis, and the mammalian target of rapamycin (mTOR) signaling pathway; mTORC1 activity affected the amount of FDG uptake within lung cancer cells 14. Moreover, AKT‐mTOR pathway activation increased PD‐L1 protein expression in NSCLC 30. Thus, a correlation between high FDG uptake and PD‐L1 protein expression might reflect AKT‐mTOR pathway activation. Additionally, FDG is actively entrapped in tumor‐related activated immune cells, such as TILs and tumor‐associated macrophages (TAMs) 31, 32. ^18^F‐FDG PET/CT could provide useful information on the metabolic state of the tumor microenvironment including tumor‐related activated immune cells and tumor cells.

We recently examined PD‐L1 protein expression in 417 surgically resected ADC specimens 28 and found it was related to smoking in ADC patients. Moreover, PD‐L1 protein expression in patients with SCC, which has a close etiological relationship with tobacco smoking, is higher than for ADC patients 33, 34. In the present study, smoking was a predictor of PD‐L1 protein expression in patients with lung cancer, especially NSCLC, suggesting PD‐L1 protein expression might be associated with smoking and oxidative stress. Furthermore, intracellular oxidative stress was upregulated in malignant carcinoma cells with high proliferative capacity 35. This report affirmed PD‐L1 protein expression might be associated with oxidative stress, and the frequency of PD‐L1 protein expression was higher in patients with both smoking history and high SUVmax than in other patients. Further in vitro or in vivo experiments are necessary to confirm these findings.

The present study had several limitations. First, this was a single institutional retrospective study and not a trial‐based correlative study; however, 579 patients were examined for associations between PD‐L1 protein expression and SUVmax in preoperative ^18^F‐FDG PET/CT. The data obtained might help identify patients with PD‐L1 expression who would benefit from antiPD‐1/PD‐L1 antibodies. The second limitation was that we investigated the association between PD‐L1 expression on tumor cells only and SUVmax. PD‐L1 protein is expressed by tumor cells and immune cells such as macrophages 36. Further study of the association between PD‐L1 protein expression on immune cells or other immune markers, such as TILs and TAMs, and SUVmax are required to assess the whole tumor microenvironment. However, the present study is the first to show a statistically significant association between SUVmax and PD‐L1 tumor protein expression in surgically resected lung cancer. The third limitation is that PD‐L1 immunohistochemistry was conducted using only one antibody and one cut‐off value. Recently, McLaughllin et al. reported that discordance was observed for PD‐L1 protein expression within a sample when using different antibodies 37. Furthermore, a positive rate using SP142 was lower than that of other antibodies including 28‐8, 22C3, and SP263. However, our previous study showed that SP142 had a higher positive rate in 40 patients with SCLC compared with other antibodies including 28‐8 and E1L3N 27. Thus, definitive antibody and cut‐off values are not established. Several antibodies and cut‐off values should be evaluated in future studies. The fourth limitation is the lack of analysis of advanced cases such as stage IV because we examined the association between PD‐L1 protein expression and SUVmax in 18 F‐FDG PET/CT using surgical specimens. The fifth limitation is that we did not exclude the cases with small tumor size to avoid the bias of partial volume effect 38. In the analysis only of the patients whose CT findings revealed a primary tumor size smaller than 20 mm, however, the results were much the same (data not shown). Further analysis only of the cases with tumor size larger than 20 mm is therefore required.

Immunohistochemistry is the main method for examining PD‐L1 protein expression, which requires biopsied or surgical specimens from patients, and invasive procedures such as transbronchial lung biopsy, CT‐guided biopsy, or surgery. However, this study suggests PD‐L1 protein expression can be predicted by medical examination and radioisotope examination, which are noninvasive or minimally invasive and can be used even if specimens from patients are unavailable. Thus, SUVmax could be used as a noninvasive tool to assess the tumor microenvironment including the frequency of PD‐L1 protein expression to predict benefit from antiPD‐1/PD‐L1 inhibitors. Standardized methods to define the cut‐off value for SUVmax should be established using studies with larger sample sizes.

In conclusion, PD‐L1‐expressing NSCLC was related to high glucose metabolism. The SUVmax in preoperative ^18^F‐FDG PET/CT was a predictor of PD‐L1 protein expression in patients with NSCLC.

## Conflict of Interest

The authors declare no conflicts of interest in association with the present study.

## Supporting information


**Figure S1.** Representative images of computed tomography (CT), ^18^F‐fluorodeoxyglucose positron emission tomography/CT (^18^F‐FDG PET/CT) and immunohistochemistry in a patient (A‐C) with programmed cell death‐ligand 1 (PD‐L1) protein expression in LCC. The maximum standardized uptake value (SUVmax) is 18.9. LCC: large cell carcinoma. Scale bar: 50 *μ*m.

**Figure S2.** The maximum standardized uptake value (SUVmax) according to programmed cell death‐ligand 1 (PD‐L1) protein expression in patients with neuroendocrine tumors (SCLC/LCNEC). There was no correlation between SUVmax and PD‐L1 protein expression (*P *=* *0.6545). SCLC: small cell carcinoma, LCNEC: large cell neuroendocrine carcinoma.

**Figure S3.** Association between the proportion of programmed cell death‐ligand 1 (PD‐L1)‐positive carcinoma cells and the maximum standardized uptake value (SUVmax) in the analyses of (A) overall (Spearman's rho = 0.4370, *P *<* *0.0001), (B) ADC/SCC/LCC (Spearman's rho = 0.4607, *P *<* *0.0001), (C) ADC (Spearman's rho = 0.3559, *P *<* *0.0001) and (D) SCC (Spearman's rho = 0.2628, *P *=* *0.0073). ADC: adenocarcinoma, SCC: squamous cell carcinoma, LCC: large cell carcinoma.

**Figure S4.** Representative images of receiver operating characteristic (ROC) curves in the analyses of (A) ADC and (B) SCC. ADC: adenocarcinoma, SCC: squamous cell carcinoma, AUC: area under curve.

**Table S1**. Association between PD‐L1 protein expression and clinicopathological factors in patients with adenocarcinoma.

**Table S2.** Association between PD‐L1 protein expression and clinicopathological factors in patients with squamous cell carcinoma.

**Table S3.** The frequency of PD‐L1 protein expression according to smoking history and SUVmax in preoperative ^18^F‐FDG PET/CT.
