# Supplementary figures and images for: Metabolic characteristics of programmed cell death‐ligand 1‐expressing lung cancer on 18F‐fluorodeoxyglucose positron emission tomography/computed tomography
Source: Cancer Med. 2017 Oct 4;6(11):2552–61. doi: 10.1002/cam4.1215 (PMC5673920; doi:10.1002/cam4.1215)

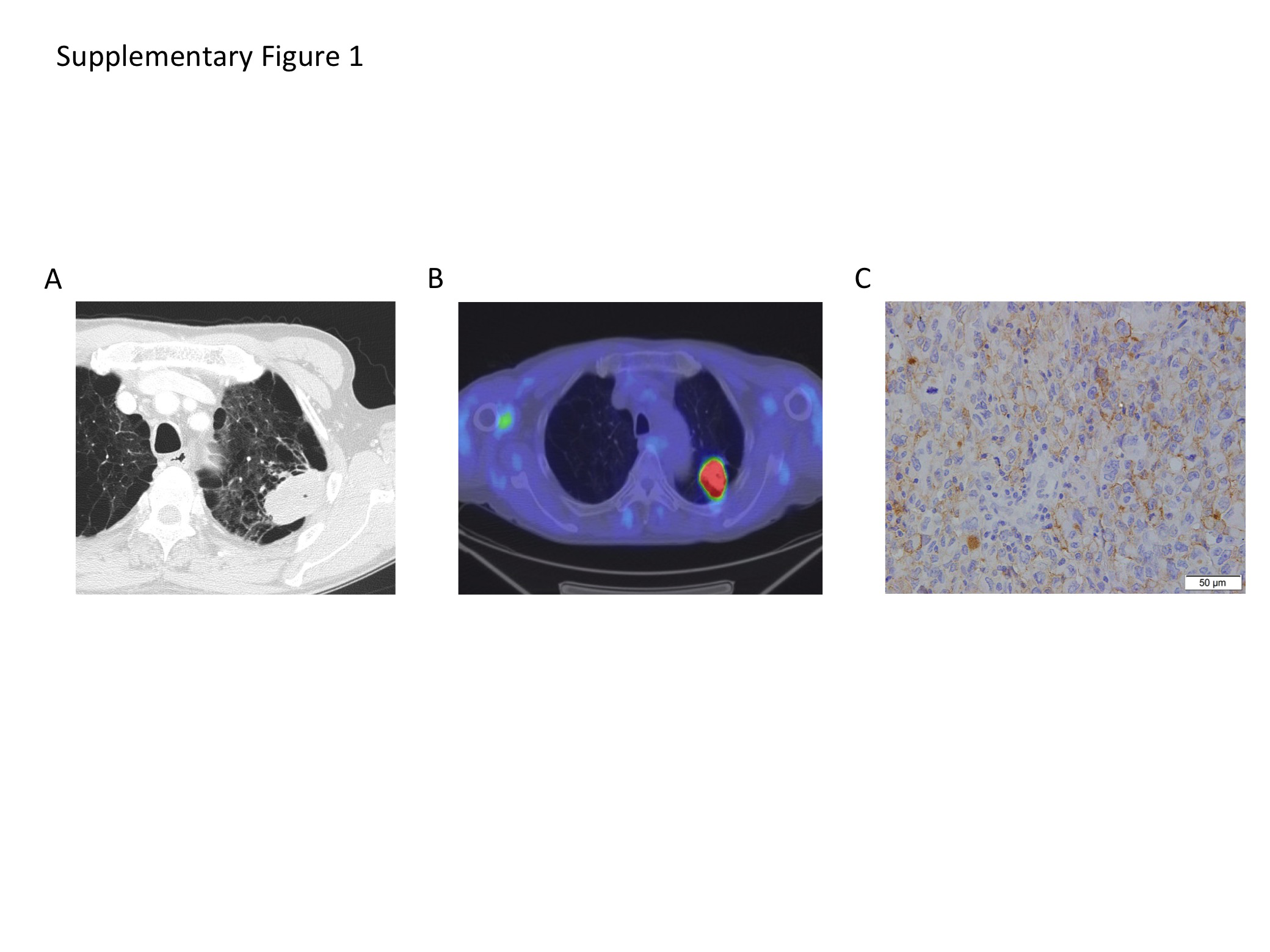

Supplement: Supplementary file 1 — Figure S1. Representative images of computed tomography (CT), 18F‐fluorodeoxyglucose positron emission tomography/CT (18F‐FDG PET/CT) and immunohistochemistry in a patient (A‐C) with programmed cell death‐ligand 1 (PD‐L1) protein expression in LCC. The maximum standardized uptake value (SUVmax) is 18.9. LCC: large cell carcinoma. Scale bar: 50 μm. [file CAM4-6-2552-s001.tif]

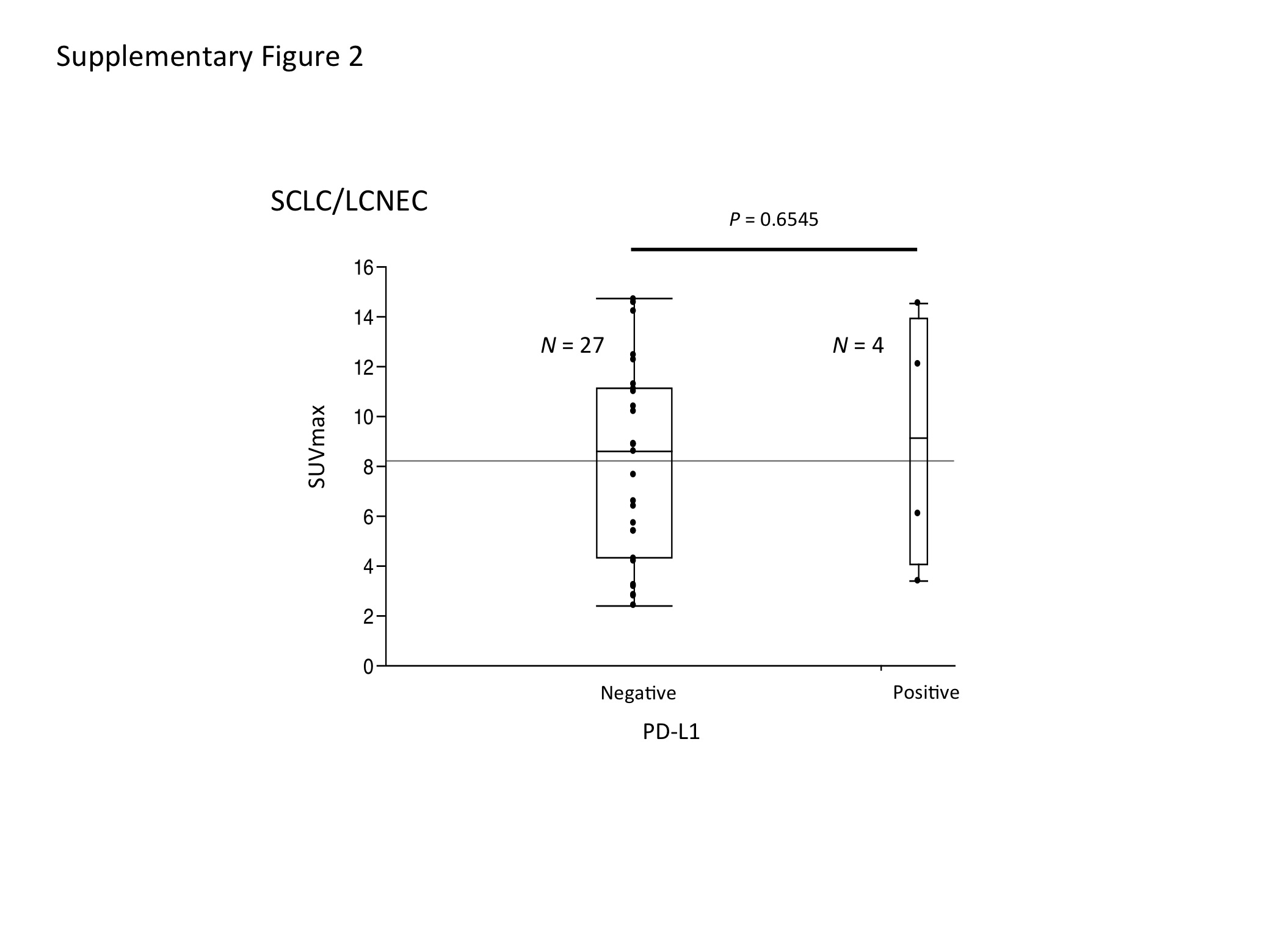

Supplement: Supplementary file 2 — Figure S2. The maximum standardized uptake value (SUVmax) according to programmed cell death‐ligand 1 (PD‐L1) protein expression in patients with neuroendocrine tumors (SCLC/LCNEC). There was no correlation between SUVmax and PD‐L1 protein expression (P = 0.6545). SCLC: small cell carcinoma, LCNEC: large cell neuroendocrine carcinoma. [file CAM4-6-2552-s002.tif]

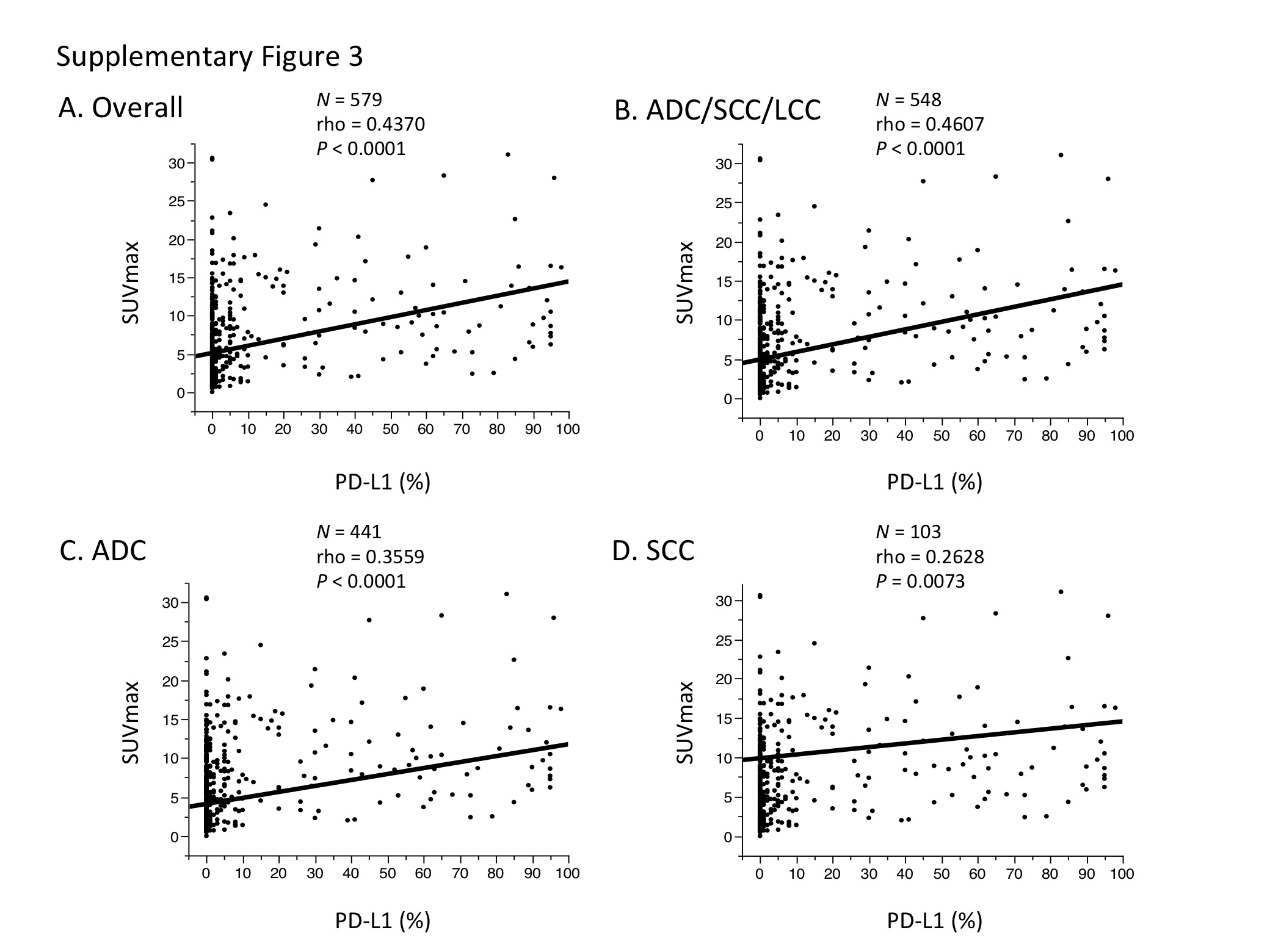

Supplement: Supplementary file 3 — Figure S3. Association between the proportion of programmed cell death‐ligand 1 (PD‐L1)‐positive carcinoma cells and the maximum standardized uptake value (SUVmax) in the analyses of (A) overall (Spearman's rho = 0.4370, P < 0.0001), (B) ADC/SCC/LCC (Spearman's rho = 0.4607, P < 0.0001), (C) ADC (Spearman's rho = 0.3559, P < 0.0001) and (D) SCC (Spearman's rho = 0.2628, P = 0.0073). ADC: adenocarcinoma, SCC: squamous cell carcinoma, LCC: large cell carcinoma. [file CAM4-6-2552-s003.tif]

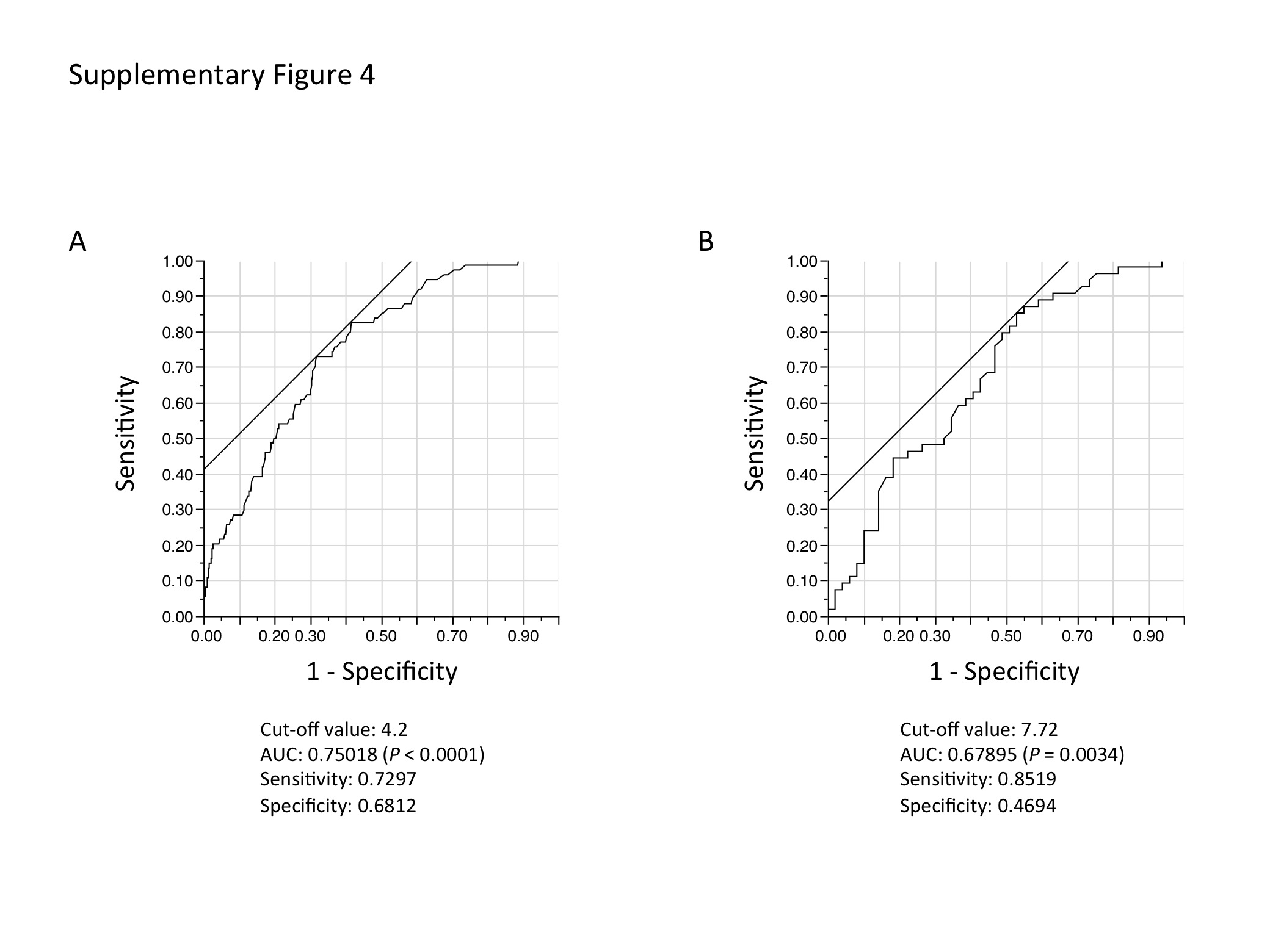

Supplement: Supplementary file 4 — Figure S4. Representative images of receiver operating characteristic (ROC) curves in the analyses of (A) ADC and (B) SCC. ADC: adenocarcinoma, SCC: squamous cell carcinoma, AUC: area under curve. [file CAM4-6-2552-s004.tif]
