# Supplementary material for: Metabolic characteristics of programmed cell death‐ligand 1‐expressing lung cancer on 18F‐fluorodeoxyglucose positron emission tomography/computed tomography
Source: Cancer Med. 2017 Oct 4;6(11):2552–61. doi: 10.1002/cam4.1215 (PMC5673920; doi:10.1002/cam4.1215)
Supplement: Supplementary file 5 — Table S1. Association between PD‐L1 protein expression and clinicopathological factors in patients with adenocarcinoma. [file CAM4-6-2552-s005.docx]

**Supplementary Table 1**. Association between PD-L1 protein expression and clinicopathological factors in patients with adenocarcinoma.

| **Factors** |  | ***N*** | **PD-L1, *N* (%)** | | ***P* value** |
| --- | --- | --- | --- | --- | --- |
|  |  |  | **Negative** | **Positive** |  |
| Age (years) | < 69 | 211 | 171 (46.6) | 40 (54.1) | 0.2533 |
|  | ≥ 69 | 230 | 196 (53.4) | 34 (45.9) |  |
|  |  |  |  |  |  |
| Sex | Male | 216 | 164 (44.7) | 52 (70.3) | < 0.0001 |
|  | Female | 225 | 203 (55.3) | 22 (29.7) |  |
|  |  |  |  |  |  |
| Smoking status | Never-smoker | 216 | 197 (53.7) | 19 (25.7) | < 0.0001 |
|  | Smoker | 225 | 170 (46.3) | 55 (74.3) |  |
|  |  |  |  |  |  |
| Grade | G1 | 252 | 245 (66.8) | 7 (9.5) | < 0.0001 |
|  | ≥ G2 | 189 | 122 (33.2) | 67 (90.5) |  |
|  |  |  |  |  |  |
| T | T1 | 285 | 250 (68.1) | 35 (47.3) | 0.0008 |
|  | ≥ T2 | 156 | 117 (31.9) | 39 (52.7) |  |
|  |  |  |  |  |  |
| N | N0 | 377 | 324 (88.3) | 53 (71.6) | 0.0005 |
|  | ≥ N1 | 64 | 43 (11.7) | 21 (28.4) |  |
|  |  |  |  |  |  |
| Stage | Ⅰ | 349 | 300 (81.7) | 49 (66.2) | 0.0044 |
|  | ≥ II | 92 | 67 (18.3) | 25 (33.8) |  |
|  |  |  |  |  |  |
| pl | Absent | 361 | 316 (86.1) | 45 (60.8) | < 0.0001 |
|  | Present | 80 | 51 (13.9) | 29 (39.2) |  |
|  |  |  |  |  |  |
| ly | Absent | 400 | 335 (91.3) | 65 (87.8) | 0.3793 |
|  | Present | 41 | 32 (8.7) | 9 (12.2) |  |
|  |  |  |  |  |  |
| v | Absent | 342 | 302 (82.3) | 40 (54.1) | < 0.0001 |
|  | Present | 99 | 65 (17.7) | 34 (45.9) |  |
|  |  |  |  |  |  |
| Histological subtype | Micropapillary/solid | 29 | 8 (2.2) | 21 (28.4) | < 0.0001 |
|  | Others | 412 | 359 (97.8) | 53 (71.6) |  |
|  |  |  |  |  |  |
| *EGFR** | Wild-type | 185 | 138 (43.5) | 47 (78.3) | < 0.0001 |
|  | Mutant | 192 | 179 (56.5) | 13 (21.7) |  |
|  |  |  |  |  |  |
| SUVmax** | Low | 270 | 250 (68.1) | 20 (27.0) | < 0.0001 |
|  | High | 171 | 117 (31.9) | 54 (73.0) |  |

*: cases whose data are available.

**: cut-off value is 4.2.

PD-L1: programmed cell death-ligand 1, pl: pleural invasion, ly: lymphatic invasion, v: vascular invasion, *EGFR*: epidermal growth factor receptor, SUVmax: the maximum standardized uptake value.
