# Supplementary material for: Metabolic characteristics of programmed cell death‐ligand 1‐expressing lung cancer on 18F‐fluorodeoxyglucose positron emission tomography/computed tomography
Source: Cancer Med. 2017 Oct 4;6(11):2552–61. doi: 10.1002/cam4.1215 (PMC5673920; doi:10.1002/cam4.1215)
Supplement: Supplementary file 6 — Table S2. Association between PD‐L1 protein expression and clinicopathological factors in patients with squamous cell carcinoma. [file CAM4-6-2552-s006.docx]

**Supplementary Table 2.** Association between PD-L1 protein expression and clinicopathological factors in patients with squamous cell carcinoma.

| **Factors** |  | ***N*** | **PD-L1, *N* (%)** | | ***P* value** |
| --- | --- | --- | --- | --- | --- |
|  |  |  | **Negative** | **Positive** |  |
| Age (years) | < 69 | 42 | 24 (49.0) | 18 (33.3) | 0.1144 |
|  | ≥ 69 | 61 | 25 (51.0) | 36 (66.7) |  |
|  |  |  |  |  |  |
| Sex | Male | 94 | 44 (89.8) | 50 (92.6) | 0.7330 |
|  | Female | 9 | 5 (10.2) | 4 (7.4) |  |
|  |  |  |  |  |  |
| Smoking history | < 30 pack years | 12 | 6 (12.2) | 6 (11.1) | 1.0000 |
|  | ≥ 30 pack years | 91 | 43 (87.8) | 48 (88.9) |  |
|  |  |  |  |  |  |
| Grade | Non-keratinizing | 20 | 9 (18.4) | 11 (20.4) | 1.0000 |
|  | Keratinizing | 83 | 40 (81.6) | 43 (79.6) |  |
|  |  |  |  |  |  |
| T | T1 | 37 | 22 (44.9) | 15 (27.8) | 0.0997 |
|  | ≥ T2 | 66 | 27 (55.1) | 39 (72.2) |  |
|  |  |  |  |  |  |
| N | N0 | 78 | 38 (77.6) | 40 (74.1) | 0.8186 |
|  | ≥ N1 | 25 | 11 (22.4) | 14 (25.9) |  |
|  |  |  |  |  |  |
| Stage | Ⅰ | 61 | 34 (69.4) | 27 (50.0) | 0.0702 |
|  | ≥ II | 42 | 15 (30.6) | 27 (50.0) |  |
|  |  |  |  |  |  |
| pl | Absent | 64 | 35 (71.4) | 29 (53.7) | 0.0711 |
|  | Present | 39 | 14 (28.6) | 25 (46.3) |  |
|  |  |  |  |  |  |
| ly | Absent | 90 | 43 (87.8) | 47 (87.0) | 1.0000 |
|  | Present | 13 | 6 (12.2) | 7 (13.0) |  |
|  |  |  |  |  |  |
| v | Absent | 59 | 31 (63.3) | 28 (51.9) | 0.3189 |
|  | Present | 44 | 18 (36.7) | 26 (48.1) |  |
|  |  |  |  |  |  |
| SUVmax* | Low | 31 | 23 (46.9) | 8 (14.8) | 0.0005 |
|  | High | 72 | 26 (53.1) | 46 (85.2) |  |

*: cut-off value is 7.72.

PD-L1: programmed cell death-ligand 1, pl: pleural invasion, ly: lymphatic invasion, v: vascular invasion, SUVmax: the maximum standardized uptake value.
