# Supplementary material for: Metabolic characteristics of programmed cell death‐ligand 1‐expressing lung cancer on 18F‐fluorodeoxyglucose positron emission tomography/computed tomography
Source: Cancer Med. 2017 Oct 4;6(11):2552–61. doi: 10.1002/cam4.1215 (PMC5673920; doi:10.1002/cam4.1215)
Supplement: Supplementary file 7 — Table S3. The frequency of PD‐L1 protein expression according to smoking history and SUVmax in preoperative 18F‐FDG PET/CT. [file CAM4-6-2552-s007.docx]

**Supplementary Table 3.** The frequency of PD-L1 protein expression according to smoking history and SUVmax in preoperative ^18^F-FDG PET/CT.

| **Histology** | **Smoking history** | **SUVmax**** | **Percentage of tumors with positive PD-L1 expression (%)** |
| --- | --- | --- | --- |
|  |  |  |  |
| Overall* | Never-smoker | Low | 2.8 (4/143) |
|  | Smoker | Low | 12.6 (18/143) |
|  | Never-smoker | High | 20.7 (17/82) |
|  | Smoker | High | 46.0 (97/211) |
| ADC/SCC/LCC | Never-smoker | Low | 2.8 (4/142) |
|  | Smoker | Low | 12.3 (17/138) |
|  | Never-smoker | High | 21.3 (17/80) |
|  | Smoker | High | 50.0 (94/188) |
| ADC | Never-smoker | Low | 2.8 (4/141) |
|  | Smoker | Low | 12.4 (16/129) |
|  | Never-smoker | High | 20.0 (15/75) |
|  | Smoker | High | 40.6 (39/96) |
| SCC | < 30 pack years | Low | 25.0 (1/4) |
|  | ≥ 30 pack years | Low | 25.9 (7/27) |
|  | < 30 pack years | High | 62.5 (5/8) |
|  | ≥ 30 pack years | High | 64.1 (41/64) |

*: ADC, SCC, LCC, SCLC and LCNEC.

**: cut-off values are 4.2, 4.2, 4.2 and 7.72 in analyses of overall, ADC/SCC/LCC, ADC and SCC, respectively.

PD-L1: programmed cell death-ligand 1, SUVmax: the maximum standardized uptake value,

^18^F-FDG PET/CT: ^18^F-fluorodeoxyglucose positron emission tomography/computed tomography, ADC: adenocarcinoma, SCC: squamous cell carcinoma, LCC: large cell carcinoma.
